# Who are the “Heroes of CRISPR”? Public science communication on Wikipedia and the challenge of micro-notability

**DOI:** 10.1177/09636625241229923

**Published:** 2024-02-28

**Authors:** Arno Simons, Wolfgang Kircheis, Marion Schmidt, Martin Potthast, Benno Stein

**Affiliations:** Technische Universität Berlin, Germany; Leipzig University, Germany; ScaDS.AI, Germany; German Centre for Higher Education Research and Science Studies (DZHW), Germany; Leipzig University, Germany; ScaDS.AI, Germany; Bauhaus-Universität Weimar, Germany

**Keywords:** CRISPR, digital humanities, genetic and reproductive technologies, history of science, innovation, notability, science communication, scientific controversies, scientific literacy, Wikipedia

## Abstract

Wikipedia’s influence in shaping public perceptions of science underscores the significance of scientists being recognized on the platform, as it can impact their careers. Although Wikipedia offers guidelines for determining when a scientist qualifies for their own article, it currently lacks guidance regarding whether a scientist should be acknowledged in articles related to the innovation processes to which they have contributed. To explore how Wikipedia addresses this issue of scientific “micro-notability,” we introduce a digital method called Name Edit Analysis, enabling us to quantitatively and qualitatively trace mentions of scientists within Wikipedia’s articles. We study two CRISPR-related Wikipedia articles and find dynamic negotiations of micro-notability as well as a surprising tension between Wikipedia’s principle of safeguarding against self-promotion and the scholarly norm of “due credit.” To reconcile this tension, we propose that Wikipedians and scientists collaborate to establish specific micro-notability guidelines that acknowledge scientific contributions while preventing excessive self-promotion.

## 1. Introduction

Wikipedia plays a central role in today’s knowledge economy and importantly as a channel for public science communication with immediate impacts on science literacy. Wikipedia articles are consistently ranked among the highest search results on virtually any search engine and widely regarded as a credible source of information by knowledge workers across the globe (Jemielniak and Aibar, 2016; Segev and Sharon, 2017; Shafee et al., 2017b). Key to Wikipedia’s success are its collaborative editing style, its free-use content, and its commitment to transparency through thorough documentation of revision histories and open discussions on talk pages. To counteract bias and edit wars, the Wikipedia community has developed policies and guidelines, which further increase trust in the platform.

Wikipedia is often compared with science (e.g. Black, 2008; Cummings, 2020). Unlike scientific publications, Wikipedia articles are very dynamic and can be edited by *anyone* at any time. Whoever clicks on the “Edit” tab of an article can immediately start editing its content and thereby becomes a Wikipedia “editor.” This flexibility allows editors to keep articles up to date by quickly incorporating current events or new findings published elsewhere. While scientific manuscripts usually go through a closed editorial and peer review before final publication, contributions to Wikipedia are published immediately but remain subject to open public review by Wikipedia editors. All changes made to a Wikipedia article are automatically documented in an open database called the “revision history,” which is easily accessible through an article’s “View history” tab. Wikipedia thus “extends the concept of revision and review [. . .and] views knowledge production as an endless process” (Benjakob and Aviram, 2018: 242). Consequently, Wikipedia has been promoted as “a potential model for more rapid and reliable dissemination of scholarly knowledge” (Black, 2008: 73).

Scientists and their institutions are becoming increasingly aware of the potential benefits of engaging with Wikipedia for at least three reasons. First, they view Wikipedia as a new channel for the communication of scientific knowledge to society and actively encourage their colleagues to contribute to and edit the platform (Heilman et al., 2011; Kincaid et al., 2021; Rosenzweig, 2006; Shafee et al., 2017b; Wyatt, 2020). Second, researchers have begun assessing the accuracy and completeness of science coverage on the platform, often concluding that more should be done to improve the quality of science content (Adams et al., 2019; Serrano-Lopez et al., 2017; Shafee et al., 2017a; Smith, 2020). Third, actors in the business of “altmetrics” have been discussing Wikipedia’s potential as a new data source for measuring scientific impact (Ortega, 2018; Zagorova et al., 2020). Wikipedia, it seems, has become an “obligatory point of passage” (Callon, 1986) for science.

This creates a self-reinforcing dynamic, where scientists and institutions increasingly seek recognition on the platform. Crucially, recognition on Wikipedia includes not only classical citations but also personal mentions. At a time when public recognition is more important to scientists and their careers than ever before, *not* being mentioned on Wikipedia can be a real issue, given that the platform significantly shapes the public’s view of science. As Samoilenko and Yasseri (2014) write, “individual scientists, their fields, and entire academic institutions, can be easily affected by the way they are represented in this important online medium” (p. 8). In other words, Wikipedia’s influence in shaping public perceptions of science underscores the significance of scientists being recognized on the platform, as it can directly impact their careers.

Wikipedians and scientists alike have already recognized the issue of “academic notability,”^1^ albeit exclusively on article level (Adams et al., 2019; Samoilenko and Yasseri, 2014; Schellekens et al., 2019). In general, on Wikipedia “notability is a test used by editors to decide whether a given topic warrants its own article.”^2^ But there is another, equally important issue to be addressed:

How does Wikipedia decide whether a scientist should be mentioned in an article that is *not* about them?

We call this the “micro-notability” problem because it applies at the sub-article level. Being mentioned as a relevant actor in an article about a science-related topic, development or innovation matters because it helps to publicly define a scientist’s achievement in the first place, often before a biographical article seems appropriate. To the best of our knowledge, scientific micro-notability has been completely overlooked so far.

Here, we study scientific micro-notability negotiations in the context of Wikipedia’s coverage of the discovery of clustered regularly interspaced short palindromic repeats (CRISPR) and CRISPR-associated (Cas) proteins. The “CRISPR revolution” involved three key steps: the discovery of unusual DNA segments in archaea and bacteria during the late 1980s and early 1990s, the recognition in the 2000s that these segments were part of a prokaryotic adaptive immunity system, and the eventual realization that these systems could be reprogrammed into “genetic scissors” used for precise gene editing by labs globally.

We selected this case because of its significant scientific and technological impact, as well as the controversy it has generated regarding recognition. As science reporter Heidi Ledford (2016a) puts it:

The history of CRISPR [. . .] has become a subject of fierce debate and a bitter, high-stakes patent battle. Researchers and institutes have been jostling aggressively to make sure that they are credited for their share of the work in everything from academic papers to news stories. (p. 342)

Ledford, having covered the CRISPR case for years, herself contributed to popularizing the innovation’s history by recognizing its “unsung heroes.” In her *Nature News* article quoted above, she intentionally acknowledged the important contributions of junior researchers who worked behind the scenes. Her piece also reads as a correction of Eric Lander’s (2016) *Cell* article “The Heroes of CRISPR,” which has been widely criticized for marginalizing the contribution of Emmanuelle Charpentier and Jennifer Doudna while prominently featuring Feng Zhang’s work at the Broad Institute, whose director happened to be Lander. In 2020, Charpentier and Doudna were awarded the Nobel Prize for Chemistry, while Zhang was not, adding to the ongoing controversies surrounding recognition, money, and influence in the CRISPR race (Cohen, 2020; Ledford, 2016b, 2022).

The significance and controversy surrounding the CRISPR innovation make it an excellent case for investigating our research question. Wikipedia has been documenting CRISPR since 2005, crafting its own narrative of the innovation history and its key figures. This account has been revised and updated over time, as the CRISPR innovation and the ensuing controversies continued to evolve. The case shows not only that editors negotiate micro-notability but how they do it, leading to the finding that argumentation is used as much as power to settle controversies and, more surprisingly, that an inherent and problematic tension seems to exist between core norms of Wikipedia and science. To address this tension, we will recommend that Wikipedians and scientists collaborate on developing specific micro-notability guidelines.

## 2. Methodology and data

Methodologically, it comes in handy that Wikipedia is not only a channel for public science communication but also a key venue for public discourse and controversy about science and innovative technologies. As many studies have shown, Wikipedia editors often argue about controversial content and try to discipline each other by referring to various guidelines and specifications such as those for referring to “reliable sources” or maintaining a “natural point of view” (Benjakob and Aviram, 2018; Gatto, 2016; Hara and Doney, 2015; Wyatt et al., 2016). Wikipedia’s editing history and conversation pages provide the opportunity to map and study such controversies both qualitatively and quantitatively (Gray et al., 2018; Moats, 2019; Weltevrede and Borra, 2016).

Wikipedia-related controversy studies are often based on actor network theory and its dictum to “follow the actors” (Fu et al., 2023; Moats, 2018; Venturini, 2010, 2012; Wyatt et al., 2016). As Costa and Cukierman (2019: 2263) emphasize, controversies on Wikipedia “are privileged occasions to observe science in the making,” and they “might translate into a variety of different actions that we should monitor closely: reverts, talk page debates, edit wars, page protections, template messages, and policy discussions.” This is exactly what we intend to do here. Building on previous work (Kircheis et al., 2023; Schmidt et al., 2023), we use the corresponding features of the Wikipedia platform, in particular the revision histories and talk pages originally developed for coordinating article improvements, to explore the dynamics of micro-notability negotiations.

**Figure 1.**
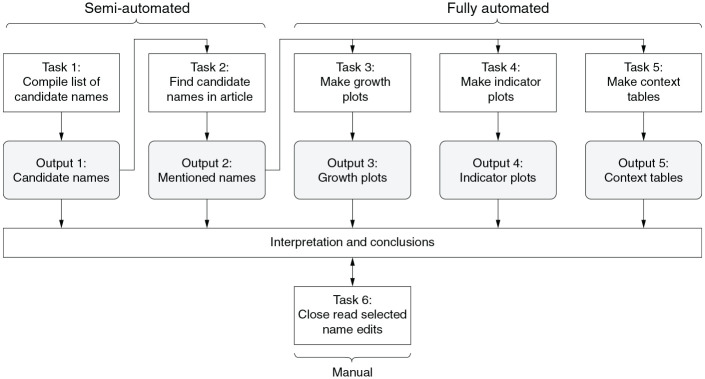
Tasks and outputs of NEA.

We introduce a novel methodological approach called Name Edit Analysis (NEA), designed to monitor name edits throughout the revision history of a Wikipedia article. Name edits include all additions and removals of researcher names, as well as changes to the scientific papers attributed to those names. NEA combines quantitative and qualitative methods as well as manual, semi-automated, and fully-automated tasks and outputs, portrayed in Figure 1. All automation was realized with custom Python scripts.^3^

Our data includes all revisions, metadata, and talk pages related to the “CRISPR” (C1) and “CRISPR gene editing” (C2) articles until 31 December 2020.^4^ C1, created on 30 June 2005, is the oldest, longest, and most general Wikipedia article on CRISPR, and it includes a separate history section. C2, created on 17 February 2019, is a spin-off from C1, with a narrower focus on CRISPR-based gene editing. C2 also includes a short history section. We downloaded 2068 revisions for C1 and 299 for C2, including metadata such as the date, editor name, and, in most cases, a short editorial comment by the editor stating the contribution of the edit. The talk pages cover article-related discussions.

To compile a list of candidate names of CRISPR researchers (Task 1), we extracted all author names from the references of all revisions of the two articles, resulting in 1255 names. In addition, we manually created a second set of 82 names based on close reading of 27 popular “historical accounts” of the CRISPR innovation, further described and analyzed in the Supplemental Material. Altogether, 1285 unique names were obtained (Output 1).

For each candidate name and for each revision of C1 and C2, the occurrence of the name in the main text body was counted (Task 2), resulting in 77 matches (initial Output 2). For each of the matched names, we manually analyzed their clustered contexts (initial Output 5) to rule out false positives. This left 36 different names mentioned at least once in C1, 9 of which are also found in C2 (final Output 2, Table S1 in the Supplemental Material).

To produce article growth plots (Task 3), the length of each revision in characters as well as the number of *unique* names and individual name *tokens* were measured for each revision of C1 and C2.^5^ In the resulting plots (Output 3), the x-axis represents time in monthly intervals, and the y-axis shows the mean article length (dashed line), the mean number of unique names (solid line), and the total number of name tokens added (green bars) and removed (orange bars).

For a nuanced quantitative aggregation of name additions and removals we developed four indicators (Task 4):

$$age=\frac{number\;of\;revisions\;since\;first\;occurrence\;of\;name}{number\;of\;all\;revisions},$$

$$endurance=\frac{number\;of\;revisions\;containing\;name}{number\;of\;all\;revisions},$$

$$prominence=\frac{total\;number\;of\;occurrences\;of\;name\;across\;all\;revisions(occurrences\;in\;the\;intro\;count\;double)}{number\;of\;revisions\;since\;first\;occurrence\;of\;name},$$

$$controversiality=\frac{sum\;of\;negative\;change\;ratios\;in\;occurrence\;frequency\;of\;name\;from\;one\;revision\;to\;the\;next}{-1}.$$

*Age* ranges from 0 to 1 and measures how early or late a name appears in the article’s history. *Age* is 1 if the name occurs in the first revision of the article and approaches 0 the later the name occurs. *Endurance* ranges from 0 to 1 and reflects how consistently a name appears across all revisions. It is 1 if the name occurs in all revisions and close to 0 if the name occurs only in a small fraction of all revisions. *Prominence* is a positive number and measures the normalized average frequency by which a name occurs during the period since its first occurrence, with extra weight given to occurrences in the lead section. *Controversiality* is a positive number that corresponds to a negative growth rate. The indicator essentially sums the weighted decrease in a name’s occurrence from one revision to the next and is expressed as a positive number for a more intuitive reading.

In the resulting indicator plots (Output 4), the circles represent the names of CRISPR researchers who appear in at least one revision of the respective article. *Prominence* is shown on the left y-axis, *Controversiality* is shown on the x-axis. The circles’ sizes indicate *endurance*. The gray scale of the circles indicates their scoring on *age*.

To support the qualitative analysis of selected name edits, we automated the generation of context tables (Task and Output 5). For each name and article a table was produced that shows all occurrence contexts of the name in all article revisions. Context is defined as up to 100 characters to the left (left context) as well as to the right (right context) of the name without crossing paragraph boundaries. Left and right contexts are clustered based on their lexical similarity to allow focusing on significant changes of each type of context across revisions while ignoring minor changes.

While we use the real names of CRISPR researchers as they appear in the articles, we have pseudonymized the usernames of Wikipedia editors to avoid baseless guessing about identities and intentions behind these names. In fact, Wikipedia encourages editors to “*choose a username that is different from your real name*, as usernames are public and cannot be made private later.”^6^ This also means that any stance or intent we wish to ascribe to an editor must be inferred from what they write or do, rather than from their name. We refer to editors using gender-neutral pronouns (they/them/theirs).

For a more detailed description of the data as well as different tasks and outputs see the Supplemental Material.

## 3. Quantitative analysis

The quantitative analysis focuses on Outputs 1–4 and provides an overview and interpretation of the dynamics of micro-notability in the two Wikipedia articles studied. It identifies relevant time periods and patterns in the positioning of names and provides initial insights into the discursive patterns surrounding these periods and positions.

### Time periods

**Figure 2.**
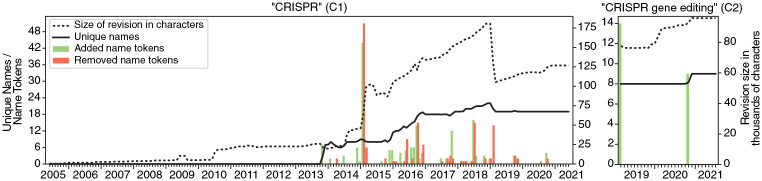
Article growth plots—the solid line shows the number of unique names of scientists mentioned in the article, while the dashed line shows the article length in characters. The green and orange bars represent the absolute number of added and removed name tokens, respectively. Please refer to online article for colour figure.

Looking at the article growth plot for C1 (Figure 2/C1), we can identify three major time periods. The first stretches from C1’s launch in June 2005 until October 2013. It is characterized by little growth and, crucially, the absence of researcher names. The little increase in length in July 2010 was due to the introduction of C1’s history section, which claimed among other things, that CRISPR was discovered in 1987 in Escherichia coli, citing a paper by Ishino et al., but not mentioning any author names in the text.

The second period runs from November 2013 to January 2019 and is characterized by a sevenfold increase in article length and intensive name editing. The period starts with the inclusion of the first seven names by Editor 1, who took them from a recently published news article in the journal *Science*, which Editor 1 references. At a time when CRISPR was hardly known outside of biochemistry, this article had been one of the first to popularize CRISPR researchers and inform a wider audience of the significance of “a potentially revolutionary genome-editing technique” (Pennisi, 2013: 833). The previous absence of similar media reporting could be an explanation for why no names of CRISPR researchers are found in earlier revisions of C1. Overall, the addition and removal of names in the first period (green and orange bars, respectively) is not evenly distributed. Three of these spikes relate to controversies reported in the qualitative analysis sections: a controversy over recognition of co-authorship (October 2016), a controversy over patents (February and March 2015), and a controversy over proper recognition versus improper promotion (July 2018). At the end of the second period, C1 contained a maximum number of 22 unique names, indicating that there must have been quite some exchange of unique names in the course of C1’s history, considering that 36 unique names appeared at some point.^7^

The third and last period starts in February 2019 with a sudden and steep decrease in article length accompanied by a less steep decrease in the number of unique names. The simple explanation for this is the transfer of several parts of C1 into a new standalone article on CRISPR gene editing, that is, C2. The rest of this period is characterized by a slow and steady increase until our cut-off date in December 2021. The only interesting addition of name tokens to mention here is the announcement in October 2020 that Doudna and Charpentier had been awarded the Nobel Prize.

For C2 the picture is simpler and less exciting. Over a period of a little more than 2 years, C2 grew within three steps of roughly equal length (dashed line), while the number of unique names (solid line) changed only once, when it increased from eight to nine in October 2020. The nine added name tokens (green bars) in October 2020 all relate to the Nobel Prize. The two winners, Doudna and Charpentier, each received four additional mentions while Šikšnys received an additional mention for not having been awarded a Nobel Prize. The absence of more edit activity and controversy in C2, as we see later, is explained by the fact that C2 was published after the key controversies had already been settled in C1.

### Positioning of names

**Figure 3.**
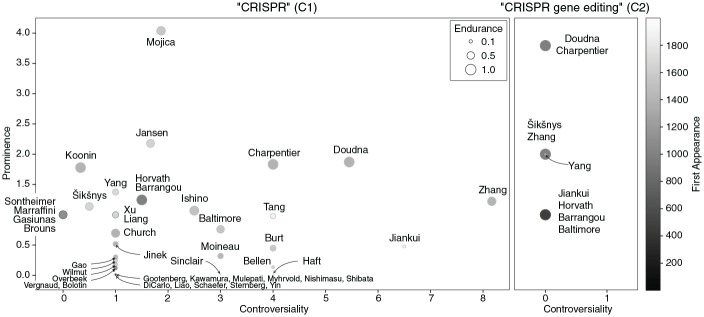
Indicator plots—distribution of CRISPR researchers along four dimensions: *prominence* (y-axis), *controversiality* (x-axis), *age* (gray scale), and *endurance* (circle size).

Figure 3 shows the indicator plots for C1 and C2 (Output 4).

The positioning of the names in C1 is diverse, but some patterns emerge. Regarding *controversiality* the majority of names is lower than 2. Only eight names are 4 or higher. Among these, Zhang, Doudna, and Charpentier have the highest levels of *prominence*. All three are credited for their contributions to the discovery of the genetic scissors, but Charpentier and Doudna are mentioned more often and more prominently, including in the article’s lead section. The positions of Zhang, Doudna, and Charpentier in Figure 3 relate to the fact that these researchers are key figures in major priority and patent disputes, whose reflection in C1 we discuss below. Haft also caught our attention because his name scores relatively high on *controversiality* while scoring very low on *endurance*, implying a lot of repeated additions and removals over a short “lifespan” in C1. As we see below, Haft was at the center of a short but intense and revealing controversy about proper recognition versus improper promotion. Regarding *prominence* only Mojica stands out in C1, scoring almost twice as high as Jansen, the second highest. Both are recognized for key contributions in earlier stages of the CRISPR innovation, including the proposal of the acronym “CRISPR.” Doudna and Charpentier take a middle position, together with Koonin, but they score higher than Zhang. There is variation regarding both *age* and *endurance* in C1: With scores of more than 0.9 on *endurance* and *age*, Barrangou, Charpentier, Doudna, Horvath, and Koonin lead. Low scores on *age*, which logically implies low scores on *endurance*, are an indication that such names are connected to contributions later in the innovation process.

The picture is again much simpler for C2. Since no names were removed, *controversiality* is zero for all nine names. There are three levels of *prominence*: Doudna and Charpentier take the top position, scoring twice as high as the names on the second level (Zhang, Yang, Šikšnys), and four times as high as those on the lowest level. Their distance is explained by the fact that both of them received four additional mentions for having been awarded the Nobel Prize (see above).

Only around half of the 36 researcher names that appeared in the two articles at some point remained mentioned by the end of 2020. All other names had by then been filtered out in the collective name editing process.

## 4. Qualitative analysis

In light of the above overview of the micro-notability negotiations, we now turn to a detailed analysis of selected name edits, along with the editorial comments and, if available, the corresponding discussions on article and user talk pages (Task 6). The analysis was performed iteratively in several rounds. The selection of name edits was informed by the above reported spikes in Figure 2 and positions in Figure 3, by interesting patterns in the context tables, as well as by intermediate results from previous rounds of close reading.

In the next two subsections, we introduce a taxonomy to categorize name edits and demonstrate its use for analyzing degrees of concern for micro-notability in negotiations of author names. Then we show how the central controversy surrounding the attribution of the discovery of CRISPR-Cas played out in Wikipedia and how it was resolved. Finally, we examine a case study, which reveals an important tension among editors between the scientific norm of “due credit” and Wikipedia’s policy of protecting against self-promotion.

### A taxonomy of name edits

A major goal of the qualitative analysis of selected name edits is to understand the editors involved in these negotiations and their perspectives. As explained in the methodology section, however, we should not infer an editor’s position or intentions from their name alone, but only from what they write or do. Editorial comments can be informative, as can exchanges between editors on talk pages. But what about the edits themselves?

A first important result of our close-reading analysis is that the name edits can be divided into three types, which tell us something about the perspectives and conflicts of the editors. All of the name edits we examined involve the attribution of a scientific contribution to a name and vice versa: *contribution ⇔ name*. Name edits can therefore be distinguished according to which part of this relationship is changed and how it is changed:

Type 1: An entire *contribution ⇔ name* relation is either added or removed.
Type 2: The *name* part of an existing *contribution ⇔ name* relation is added or removed while the *contribution* part remains in place.
Type 3: The *contribution* part of an existing *contribution ⇔ name* relation is changed semantically, that is, its meaning is changed, while the *name* part remains in place.

This taxonomy helps to talk about what exactly has been changed in a name edit and what we can infer from that change about an editor’s reflection and problematization of micro-notability. Type 1 edits do not reveal how much an editor values micro-notability, as it remains unclear whether the editor’s decision to insert or delete the *contribution ⇔ name* is due to the relation itself or a more neutral goal such as lengthening or shortening the corresponding passage in the article. In a Type 2 edit, however, an editor operates on an existing *contribution* by explicitly coupling it with or decoupling it from a *name*, thereby problematizing the previous absence or presence of the name, respectively. When Type 2 edits take the form of name swaps, that is, re-attributions of scientific credit, they signal an even stronger concern for micro-notability. In a Type 3 edit, an editor changes the *meaning* of the scientific *contribution* and thus possibly the amount of credit attributed to a *name* , again suggesting a strong interest in micro-notability.

In the remainder of this chapter, we will use this taxonomy, explicitly or implicitly, to interpret the conflicts between editors and draw inferences about the social dynamics of micro-notability negotiations.

### Authorship and recognition

Reflection and problematization of micro-notability often occur in situations in which researchers are mentioned in close connection to sources they co-authored. Using our taxonomy, we show through two examples (Table 1) and the associated discussions that consideration of author hierarchies and recognition of contributions play a role even in seemingly unremarkable edits and can become a source of conflict.

**Table 1.** Type 2 edits problematizing micro-notability in relation to scientific status hierarchies.

Date	Name edit	Type	Comment (editor)
20 January 2016	Jinek (?) **In 2012, a group including both Doudna and Charpentier** combined tracrRNA and spacer RNA into a “single-guide RNA” molecule that, mixed with Cas9, could find and cut the correct DNA targets. **Their study** Jinek et al proposed that such synthetic guide RNAs could be used for gene editing.[SOURCE]	Type 2 removal (Jinek), Type 2 addition (Doudna/ Charpentier)	minor clarification (Editor 2)
22 June 2016	In 2007 Barrangou, Horvath (food industry scientists at Danisco) and others **Moineau’s group at Université Laval (Canada)** showed that they could alter the resistance of Streptococcus thermophilus to phage attack with spacer DNA.[SOURCE]	Type 2 addition (Moineau)	→History (Editor 3)

In the “Name Edit” column, the added text is in bold and the removed text is crossed out. “[SOURCE]” stands for a reference.

In January 2016 “Jinek (?)” was replaced by “a group including both Doudna and Charpentier” in a sentence that reported a particular study and publication. Since this Type 2 name swap was declared as “minor clarification” in the editorial comment, one may ask what exactly needed to be clarified here. The paper was co-authored by Martin Jinek, Krzysztof Chylinski, Ines Fonfara, Michael Hauer, Jennifer Doudna, and Emmanuelle Charpentier, in this order. As is common in biochemistry and related fields the order of name encodes information about the roles and the status of the co-authors. Those who conduct the central experiments are usually listed first, while those who manage the laboratories where the experiments are conducted are listed last. We assume that a concern about these roles and their associated hierarchies was at play here, that the “clarification” really was about shifting the attention from the experimenter (Jinek) to the lab directors (Doudna, Charpentier).^8^

The second example shows how the descriptor “others” was replaced by the name of another co-author, Sylvain Moineau, and his group (Type 2 addition). In fact, this edit is part of a whole series of mainly Type 2 edits dealing with the notability of particular co-authors of an influential paper, which provided experimental evidence that CRISPR works as an adaptive immune system. This negotiation, which we reproduce in Table S5 in the Supplemental Material, was largely about whether or not Moineau should be mentioned along with first author Rodolphe Barroungou and last author and lab director Philippe Horvath. What is interesting about this case is that although Moineau is third to last in the list of co-authors, he is himself a lab director. Throughout the debate, his name was added and removed several times in succession, until finally it was decided not to mention any of the co-authors and only to describe the work only in the passive voice (“In 2007, the first experimental evidence [. . .] was published”).

At the height of this debate on 29 October 2016, two editors engaged in a 20-minute battle. Their comments show a clash of two key norms that seem to underlie micro-notability decisions in Wikipedia in general. Two minutes after Editor 4 reinstated Moineau (whose name had been removed 4 months earlier), Editor 5 removed all three names and stated in his comment that “we don’t care about this attribution,” drawing a discursive line between the other editor and a “we,” seemingly implying something along the lines of “we, the (leading) Wikipedia editors, don’t care.” This was countered by Editor 4’s comment, “lol—‘we’ like you! But that’s okay!” to instead single out and isolate Editor 5 as one of many editors. Eventually, Editor 5 removed the names again and raised the “promotion” flag in the comment, representing the policy that Wikipedia is “not a soapbox, a battleground, or a vehicle for propaganda, advertising, and self-promotion.” What seems to clash here is the concern for the scholarly norm of due credit, as expressed by Editor 4, and the Wikipedia policy of rejecting promotion or advertising, as cited by Editor 5. The way scientific recognition and promotion are juxtaposed here suggests some tension between the two concerns, which we will return to below.

Overall, these edits, all of which we have reproduced in Table S5 in the Supplemental Material, show not only how vigorously the micro-notability of co-authors can be debated, but also that concerns about scientific status hierarchies are not welcomed by all Wikipedia editors.

### Priority and differentiation

The question of primary attribution for the discovery of the CRISPR-Cas mechanism has been the subject of controversy, including on Wikipedia. Editors debated both who should be credited for this invention and whether or not ongoing patent applications should be discussed.

It seems that the issue of priority could be resolved by an increasing differentiation of claims from 2013 onwards. Initially, the claim that “CRISPR was first detected in human cells by. . .” has been attributed and re-attributed (by Type 2 swaps) to George Church, Feng Zhang, and Jennifer Doudna in four different combinations: Church alone, Zhang and Church, Doudna and Church, and Zhang alone (Table S6 in the Supplemental Material). Then in 2016, the editors began distinguishing between the first applications of CRISPR “in humans” and “in bacteria,” attributing the former to Zhang and Church and the latter to Doudna and Charpentier (Type 3 edits). In parallel, Virginijus Šikšnys, Barrangou, and Horvath had begun receiving credit for having shown that Cas9 can be reprogrammed to target a site of choice by changing the sequence of its crRNA. More and more, editors agreed to attribute the most important invention of genetic scissors to Doudna and Charpentier, devoting an entire paragraph to a detailed description of their invention, also acknowledging the contribution of Šikšnys, Barrangou, and Horvath. The last revision of C1 under investigation (30 December 2020) mentions only in a subsequent paragraph that “Groups led by Feng Zhang and George Church simultaneously published descriptions of genome editing in human cell cultures using CRISPR-Cas9 for the first time.” It should be noted that an earlier version had clarified that these groups had carried out their work “simultaneously.” In other words, through a series of successive Type 1, 2, and 3 edits, what was originally a single and contested priority claim gradually became a multiplicity of claims that could be clearly attributed to specific research groups. All of the name edits mentioned here and many more can be examined in detail in Table S6 in the Supplemental Material.

The reflection of ongoing patent disputes in C1 in 2014 was not solved through differentiation but rather through power. It includes two notable episodes partly responsible for spikes in the growth plot (tall bars on the respective dates) and indicator plots (relatively high controversy scores of Zhang, Doudna, and Charpentier). First, on 25 February 2015, a brief edit war broke out between Editor 6, who repeatedly proposed a significant expansion of the short existing patent section (Type 1 additions), and a number of other editors who rejected and reversed this change (Type 1 removals). The proposed expansion consisted of a 600-word, barely readable paragraph comparing the patent applications of Zhang’s team with those of the Doudna and Charpentier team, and clearly in favor of the former team. While Zhang’s team had “15 US and European Patent Applications ALLOWED,” the team of Doudna and Charpentier had only “two pending patent applications,” both of which had been rejected by “Third Party Observations,” and so on. Editors 5, 7, 8, and 9 repeatedly retracted and criticized this paragraph, while Editor 6 brought it back repeatedly, a total of three times in a row. The opposing editors mainly invoked two Wikipedia policies, “no original research” and “undue weight,” the latter of which demands that editors “should not give minority views or aspects as much of or as detailed a description as more widely held views or widely supported aspects.”^9^ The second patent-related, equally brief edit war took place on 25 March 2015, between Editor 10 on one side and Editors 5, 8, and 11 on the other. Editor 10 repeatedly proposed the (Type 1) addition of a new paragraph mentioning two patent applications, one by “Zhang as sole inventor,” the other by “Doudna and others.” Whereas Editor 10 insisted that patents are valid sources, the other editors forcefully explained that “Patents are *not* primary sources” (comment by Editor 13). The short interlude was declared an “edit war” by Editor 5 and discussed on the talk pages of both Editor 10 and Editor 5, leading to mutual accusations and threats. Eventually, Editor 10 gave up. Taken together, these edits show that CRISPR-related patent disputes were also brought to Wikipedia, but were quickly and effectively suppressed through the use of various measures in one-to-many confrontations.

### Proper recognition versus improper promotion?

Finally, we examine a case that not only illustrates the role of policies in resolving micro-notability conflicts, but also points to an interesting tension between the scientific norm of “due credit” and Wikipedia’s policy of protecting against self-promotion. In July 2018 (tall bars in Figure 2/C1), Editor 12 attempted to defend the inclusion of two priority claims in the history section of C1. The first was that “Haft, et al. clarified that [. . .most CRISPR-Cas protein families. . .] represented markers for distinct CRISPR system subtypes.” The second claim stated that “Tang had actually shown [. . .] that CRISPR repeat regions [. . .] were transcribed as RNA and systematically cleaved.” However, other editors vehemently rejected both claims, leading to a back-and-forth editing of repeated Type 1 additions by Editor 12 and Type 1 removals by Editor 13, 14, and 15. The latter explicitly referred to this as an “edit war.” Simultaneously, a heated debate about these edits erupted on C1’s talk page.

This controversy sheds light on three significant issues. First, it highlights the importance of scientists being mentioned on Wikipedia within the context of innovation histories. Editor 12, who claimed to be a CRISPR-scientist, argued that “excitement about CRISPR makes the history section vital to the article” (July 13) and that “[t]he edit history of the CRISPR article reflects intense interest in controlling the attribution of credit” (July 14). Strikingly, Editor 12 even speculated on the existence of a causal relationship between citations in C1’s history section and citations in secondary sources:

That omission [of the Haft et al. paper] seems both unfortunate and self-perpetuating, since the Wikipedia article’s lack of any mention of [that paper] [. . .] probably affects today’s secondary sources (July 1).

Second, this case highlights the fact that policies designed to resolve or prevent conflicts can sometimes become controversial themselves. Whenever other editors attempted to block Editor 12’s edits by citing “reliable sources,” “self-promotion,” or “conflict of interest,” Editor 12 would challenge the applicability of these policies. For instance, when questioned about the appropriateness of citing oneself, Editor 12 argued that it was “allowed within reason” and that they followed this policy (July 13).^10^ The other editors responded to this by either reinforcing the policies in question or proposing changes to them. When asked to provide a secondary source, Editor 12 suggested a review article but was once again rejected. The other editors argued, first, that the source was not an “independent secondary source” because Editor 12 had claimed to be one of its co-authors, and second, that the source was “a bit old.” In addition, they stated that even if the source was acceptable, the proposed edit was “far too detailed” and not appropriate for the history section (July 11). This demonstrates the extent to which editors can utilize platform policies to support or oppose the credibility and micro-notability of sources and authors.

Finally, this case exposes the tense renegotiation of the roles of editors versus scientists, particularly on the talk page. Editor 15 repeatedly attempted to leverage their personal expertise to their advantage, arguing that “[s]econdary sources can be notoriously bad at crediting people who did not do much to publicize their work” (July 6) and that “review articles [are] a special standard for medical information [. . .which] does not apply to [. . .] the history section of a biochemistry article” (July 28). However, the other editors maintained that, as Wikipedia editors, it was not their place to decide whether scientists were given appropriate credit in review articles. As Editor 13 put it, “[a]s editors of Wikipedia articles, it’s not up to us to decide whether certain scientists have been given the appropriate credit in review articles” (July 6). The tone between both sides eventually escalated, with Editor 5 writing, “[w]e all get it that you write science articles every day. You do that in articles with your name on them. Wikipedia is not like that. [. . .] So for about the bazillionth time—no” (July 28). In the end, Editor 12 reintroduced only the second priority claim and described the Tang study, an edit that was eventually accepted by other editors.

## 5. Conclusion and discussion

We defined scientific *micro-notability* as the previously overlooked problem of how Wikipedia decides which scientists to mention in an article. To study the negotiation of micro-notability, we introduced a novel approach called NEA, a digital method which combines quantitative and qualitative strategies. We used NEA to systematically track mentions of researcher names in the revision history of two key Wikipedia articles on the CRISPR revolution, C1 and C2. Wrapping up, we now present and discuss our five main conclusions.

First, the negotiation of micro-notability in the two studied articles underwent periods of varying name edit activity. The most intense name editing occurred between November 2013 and February 2019, a period throughout which we observed a number of controversies on co-authorship mentions, on priority and patent disputes and on proper recognition versus improper promotion of scientific achievement. This “hot” period was preceded (in C1) as well as followed (in C1 and C2) by comparably “cold” periods of less intense and less controversial name editing. The timing of outside events played a key role here. C1 witnessed key events in the CRISPR innovation in real-time, including the invention of the genetic scissors in 2012 and an aggressive legal and media battle over priority claims and patents in 2015/2016, both of which were controversially reflected in the “hot” period of C1. C2, in contrast, was published in February 2019 after the major scientific and technological breakthroughs had been made and after key controversies had already been settled in C1.

Second, when negotiating micro-notability, editors use argumentation as well as power. To a certain degree, Wikipedia policies employed to justify name edits were used selectively, and sometimes the use of a policy itself was up for debate. Also, some of the studied controversies took the form of one-to-many confrontations and were “solved” only because a single contester gave up against other editors without really accepting their reasoning. We further observed that certain editors, such as Editors 5 and 13, who seemed to be more active than others, were also more dominant and successful in stating their positions. This suggests the existence of implicit hierarchies among editors, which should be studied further.

Third and rather surprisingly, there seems to be an inherent and problematic tension between key norms of Wikipedia and science. First of all, editors fought interpretations of what it means to be a sound Wikipedia editor. In our last case study, an editor who identified as a CRISPR researcher and wanted their own contributions to be recognized in C1 was accused of self-promotion by other editors in a one-to-many confrontation. In this episode we observed that the opposing editors used what might be called “reversed” boundary work (cf. Gieryn, 1983) to demarcate the work and role of Wikipedia editors from the work and roles of scientists, culminating in the statement: “Wikipedia is not like that.” More importantly, this episode revealed a tension between Wikipedia’s principle of safeguarding against self-promotion and the scientific norm of granting “due credit” to contributors. Strikingly, the editor accused of self-promotion expressed the fear that *not* being mentioned on Wikipedia could negatively influence subsequent citation dynamics, which is a serious issue that has been little researched. Decisions about micro-notability and the inclusion of scientific literature in Wikipedia may lead to feedback loops from Wikipedia into scientific text production, as studied by Thompson and Hanley (2018), and ultimately affect the social structure of scientific communities.^11^ Wikipedians should be aware of such dynamics and reflect their implications also in terms of their own responsibility for recognizing scientific achievements in a fair way.

Fourth and directly related, we see an unfortunate lack of guidance for treating micro-notability. In the absence of such guidance, editors used policies such as “reliable sources,” “(self-)promotion,” and “undue weight” to attack opponents’ name edits and/or to defend their own name edits. But these policies cannot grasp all aspects at stake in micro-notability negotiations.

Although some policies can be invoked to justify name removals, there is currently no explicit policy in place that provides guidance to editors on how to make informed decisions regarding the *inclusion* of achievements and names within an article, with the aim of enhancing the overall balance of names already mentioned. However, this is precisely the issue at stake in the broader perspective of recognition as a fundamental institutional norm within science, as articulated by Merton (1957). If the Wikipedia community accepts that micro-notability is an issue worth addressing, it should consider developing specific policies for guiding both the notability of names already on the table and for finding the names that are notable but still missing. Developing such guidelines could be a joint effort between Wikipedians and scientists, and help strike a balance between protecting Wikipedia from (self-)promotion and giving scientists due credit and recognition.

Fifth and finally, our analysis shows that NEA is a promising approach to studying micro-notability negotiations; but there is room for improvement. Building on our observation of editor power, future NEA studies could try to integrate information about potential hierarchies among editors, for example, in terms of their editing activity and decision power.^12^ A logical next step would be to apply NEA to more cases and to explore additional avenues for automation, for example, through the use of large language models (LLMs), for example, for the automated classification of name edits.

More generally and prospectively, it remains to be seen whether and how Wikipedia’s importance will be affected by the widespread use of LLMs. Most if not all current LLMs have been trained on Wikipedia content and they are perfectly capable of generating text “that seems a lot like it came from Wikipedia” (Deckelmann, 2023). While LLMs could help improve the editing process there is also a possibility that future LLMs could be able replace human deliberation of sources by automating summaries of the scientific literature, eventually replacing Wikipedia altogether (Gertner, 2023). Deckelmann (2023) remains hopeful, stressing that Wikipedia is trustworthy “*because* it is created, debated, and curated by people.”

## Supplemental Material

sj-docx-1-pus-10.1177_09636625241229923 – Supplemental material for Who are the “Heroes of CRISPR”? Public science communication on Wikipedia and the challenge of micro-notability Supplemental material, sj-docx-1-pus-10.1177_09636625241229923 for Who are the “Heroes of CRISPR”? Public science communication on Wikipedia and the challenge of micro-notability by Arno Simons, Wolfgang Kircheis, Marion Schmidt, Martin Potthast and Benno Stein in Public Understanding of Science

## Notes

1. https://en.wikipedia.org/wiki/Wikipedia:Notability_(academics)

2.
https://en.wikipedia.org/wiki/Wikipedia:Notability

3. You can access all our scripts here: https://github.com/webis-de/PUS-23

4. You can access all our data files here: https://doi.org/10.5281/zenodo.10507820

5. To clarify, if a given revision contains the name “Smith” twice and the name “Schmitt” once, but no other names, then it contains *two unique* names (“Smith” and “Schmitt”) but *three* name *tokens* (“Smith,” “Smith,” and “Schmitt”).

6. https://en.wikipedia.org/w/index.php?title = Special:CreateAccount&returnto = Help%3AGetting + started

7. The steep increase in article length from July 2014 onward is partly due to the inclusion of information from more cited sources (Schmidt et al., 2023).

8. Ironically, Jinek, interviewed by Ledford (2016a: 342) about his thoughts on the fact that “graduate students and postdocs are often overlooked” in the CRISPR innovation, said: “That’s just how the system works, and I accept my role in this system [. . .] But yeah, it’s something you can’t help but think about.”

9.
https://en.wikipedia.org/wiki/Wikipedia:Neutral_point_of_view#Due_and_undue_weight

10. We already encountered a similar situation above, when Editor 10 insisted on his view that patents are valid sources.

11. Representational gaps resulting from these dynamics should therefore also be considered when using Wikipedia references for altmetrics (cf. own work).

12. Figures S2 and S3 in the Supplemental Material could be part of such information. We included them after we had already finalized our analyses, but hope they can inspire future NEA studies. Also we are grateful to our anonymous reviewers for having pointed us in this direction.
