# Supplementary material for: Who are the “Heroes of CRISPR”? Public science communication on Wikipedia and the challenge of micro-notability
Source: Public Underst Sci. 2024 Feb 28;33(7):918–34. doi: 10.1177/09636625241229923 (PMC11504141; doi:10.1177/09636625241229923)
Supplement: sj-docx-1-pus-10.1177_09636625241229923 – Supplemental material for Who are the “Heroes of CRISPR”? Public science communication on Wikipedia and the challenge of micro-notability [file sj-docx-1-pus-10.1177_09636625241229923.docx]

Supplemental Material to the article "Who are the ‘Heroes of CRISPR’? Public Science Communication on Wikipedia and the Challenge of Micro-Notability"

Arno Simons (Technische Universität Berlin)
Wolfgang Kircheis (Leipzig University)
Marion Schmidt (German Centre for Higher Education Research and Science Studies, DZHW)
Martin Potthast (Leipzig University and ScaDS.AI)
Benno Stein (Bauhaus-Universität Weimar)

# Introduction

This supplement to the article "Who are the ‘Heroes of CRISPR’? Public Science Communication on Wikipedia and the Challenge of Micro-Notability", published in *Public Understanding of Science*, contains three types of additional information:

1. Additional information on data
2. Additional information on the tasks and outputs of Name Edit Analysis
3. Additional analyses of data

# Additional Information on Data

## Selection of Wikipedia Articles

We selected C1 and C2 out of 17 Wikipedia articles containing either "crispr" or "cas" in their title:

1. "Anti-CRISPR"
2. "Cas1"
3. "Cas2"
4. "Cas3"
5. "Cas4"
6. "Cas9"
7. "CRISPR"
8. "CRISPR_activation"
9. "CRISPR/Cas_Tools"
10. "CRISPR/Cpf1"
11. "CRISPR-Display"
12. "CRISPR_gene_editing"
13. "CRISPR_interference"
14. "CRISPR_Therapeutics"
15. "Genome-wide_CRISPR-Cas9_knockout_screens"
16. "No-SCAR_(Scarless_Cas9_Assisted_Recombineering...)"
17. "The_CRISPR_Journal"

Only "CRISPR" (C1) and "CRISPR_gene_editing" (C2) treated the topic from a considerable historical perspective and were thus selected for a full revision history analysis.

## Selection of the Cut-Off Date

The selection of our cut-off date, December 31, 2020, is mainly owed to the fact that data collection was completed in the spring of 2021.

## Historical Accounts Corpus

To provide additional context for our overall analysis, we gathered a corpus of popular "historical accounts" of the CRISPR innovation from Google Scholar, including academic books and non-academic sources such as media articles and gray literature. Using the query terms "crispr history" and "crispr development", we downloaded sources focused on CRISPR's innovation history until we exhausted relevant results, totaling 27 accounts after excluding irrelevant sources:

Adli M (2018) The CRISPR tool kit for genome editing and beyond. *Nature Communications* 9(1): 1911.

Barrangou R and Horvath P (2017) A decade of discovery: CRISPR functions and applications. *Nature Microbiology* 2(7): 1–9.

Broad Institute (2015) CRISPR Timeline. Available at:<https://web.archive.org/web/20151104142408/https://www.broadinstitute.org/what-broad/areas-focus/project-spotlight/crispr-timeline> (accessed 30 November 2020).

Chen S, Yao Y, Zhang Y, et al. (2020) CRISPR system: Discovery, development and off-target detection. *Cellular Signalling* 70: 109577.

Doudna JA and Charpentier E (2014) The new frontier of genome engineering with CRISPR-Cas9. *Science* 346(6213): 1258096.

Han W and She Q (2017) CRISPR history: discovery, characterization, and prosperity. In: *Progress in Molecular Biology and Translational Science*. Elsevier, pp. 1–21.

Hsu PD, Lander ES and Zhang F (2014) Development and applications of CRISPR-Cas9 for genome engineering. *Cell* 157(6). Elsevier: 1262–1278.

Ishino Y, Krupovic M and Forterre P (2018) History of CRISPR-Cas from Encounter with a Mysterious Repeated Sequence to Genome Editing Technology. *Journal of Bacteriology* 200(7).

Javed MR, Sadaf M, Ahmed T, et al. (2018) CRISPR-Cas system: history and prospects as a genome editing tool in microorganisms. *Current microbiology* 75(12). Springer: 1675–1683.

Lander ES (2016) The Heroes of CRISPR. *Cell* 164(1–2): 18–28.

Lau V and Davie JR (2017) The discovery and development of the CRISPR system in applications in genome manipulation. *Biochemistry and Cell Biology* 95(2): 203–210.

Ledford H (2016a) The unsung heroes of CRISPR. *Nature News* 535(7612): 342.

Ledford H (2016b) Titanic clash over CRISPR patents turns ugly. *Nature* 537(7621).

Ledford H and Callaway E (2020) Pioneers of revolutionary CRISPR gene editing win chemistry Nobel. *Nature* 586(7829): 346–347.

Liang P, Zhang X, Chen Y, et al. (2017) Developmental history and application of CRISPR in human disease. *The Journal of Gene Medicine* 19(6–7). Wiley Online Library: e2963.

Marraffini LA (2015) CRISPR-Cas immunity in prokaryotes. *Nature* 526(7571): 55–61.

Mojica FJM and Garrett RA (2013) Discovery and Seminal Developments in the CRISPR Field. In: Barrangou R and van der Oost J (eds) *CRISPR-Cas Systems: RNA-Mediated Adaptive Immunity in Bacteria and Archaea*. Berlin, Heidelberg: Springer, pp. 1–31.

Mojica FJM and Montoliu L (2016) On the Origin of CRISPR-Cas Technology: From Prokaryotes to Mammals. *Trends in Microbiology* 24(10): 811–820.

Mojica FJM and Rodriguez‐Valera F (2016) The discovery of CRISPR in archaea and bacteria. *The FEBS Journal* 283(17): 3162–3169.

Morange M (2015a) What history tells us XXXIX. CRISPR-Cas: From a prokaryotic immune system to a universal genome editing tool. *Journal of biosciences* 40(5): 829–832.

Morange M (2015b) What history tells us XXXVII. CRISPR-Cas: The discovery of an immune system in prokaryotes. *Journal of Biosciences* 40(2): 221–223.

Pennisi E (2013) The CRISPR craze. *Science* 341.

Riordan SM, Heruth DP, Zhang LQ, et al. (2015) Application of CRISPR/Cas9 for biomedical discoveries. *Cell & Bioscience* 5(1): 33.

Sherkow JS (2015) Law, history and lessons in the CRISPR patent conflict. *Nature biotechnology* 33(3): 256–257.

van Erp PB, Bloomer G, Wilkinson R, et al. (2015) The history and market impact of CRISPR RNA-guided nucleases. *Current opinion in virology* 12: 85–90.

Zhang C, Quan R and Wang J (2018) Development and application of CRISPR/Cas9 technologies in genomic editing. *Human Molecular Genetics* 27(R2): R79–R88.

Zhang F (2019) Development of CRISPR-Cas systems for genome editing and beyond. *Quarterly Reviews of Biophysics* 52.

# Additional Information on the Tasks and Outputs of Name Edit Analysis

## Task 2/ Output 2: Name Search

When searching for the occurrence of the 1285 unique names compiled in Output 1, we added non-accent variants for all names with accents as well as "name atoms" for all names containing spaces or hyphens. Name atoms were generated by splitting names into their distinct parts before and after a space or hyphen. For example, the name "Doe-Smith" would have been splitted into two distinct atoms: "Doe" and "Smith".

Name search was done over all revisions and candidate names while ignoring names that appear in side panels, info boxes, and the reference section. This ensured that only the names mentioned in the main article text were counted. We did not exclude in-text citations since Wikipedia only uses numerical in-text citations that do not contain any names.

Our analysis considered family names only, since the naming of researchers within Wikipedia articles does not always include first names. To the best of our knowledge, each unique family name in C1 or C2 belongs to the same person, but this is a fact we could only establish manually.

### Names in C1 and C2

Table S1 presents Output 2, the names of 36 researchers found in the two Wikipedia articles "CRISPR" (C1) and "CRISPR gene editing" (C2), together with the first day of their appearance in C1 and C2, respectively

| Name | First in C1 | First in C2 |
| --- | --- | --- |
| Baltimore | 2015-12-18 | 2019-02-17 |
| Barrangou | 2013-11-26 | 2019-02-17 |
| Bellen | 2016-10-13 |  |
| Bolotin | 2016-01-19 |  |
| Brouns | 2016-06-22 |  |
| Burt | 2015-12-21 |  |
| Charpentier | 2013-11-26 | 2019-02-17 |
| Church | 2013-11-12 |  |
| DiCarlo | 2017-10-04 |  |
| Doudna | 2013-11-26 | 2019-02-17 |
| Gao | 2014-07-23 |  |
| Gasiunas | 2016-09-28 |  |
| Gootenberg | 2019-10-26 |  |
| Haft | 2018-07-01 |  |
| He | 2018-11-27 | 2019-02-17 |
| Horvath | 2013-11-26 | 2019-02-17 |
| Ishino | 2015-09-30 |  |
| Jansen | 2016-08-09 |  |
| Jinek | 2013-11-26 |  |
| Koonin | 2013-11-26 |  |
| Liang | 2017-11-20 |  |
| Marraffini | 2016-06-22 |  |
| Moineau | 2014-07-25 |  |
| Mojica | 2016-01-19 |  |
| Mulepati | 2016-04-29 |  |
| Myhrvold | 2019-10-26 |  |
| Sontheimer | 2016-06-22 |  |
| Sternberg | 2019-01-29 |  |
| Tang | 2018-07-01 |  |
| Van der Oost | 2018-03-30 |  |
| Vergnaud | 2016-01-19 |  |
| Xu | 2017-11-20 |  |
| Yang | 2016-11-15 | 2019-02-17 |
| Yin | 2018-04-27 |  |
| Zhang | 2014-02-05 | 2019-02-17 |
| Šikšnys | 2016-09-28 | 2020-10-12 |

**Table S1**: Names of 36 researchers found in the two Wikipedia articles "CRISPR" (C1) and "CRISPR gene editing" (C2), together with the first day of their appearance in C1 and C2, respectively.

## Task 3/ Output 3: Growth Plots

To keep the article growth plots readable and informative, we excluded data related to copyright infringements (nine in C1, zero in C2),^^[[1]](#footnote-1)^^ one-time layout mess-ups, such as repetitions of the whole article (three in C1, zero in C2), and cases identified as vandalism by bots or editors using vandalism-detection tools such as Twinkle or Huggle (ten in C1, zero in C2).

## Task 5/ Output 5: Context Tables

A context table has as many rows as there are individual mentions of the name across all revisions of the article. If a name occurs n times in a given revision, n rows are added. Each row contains a column called "left", showing up to 100 characters found immediately to the left of the name in that revision but still within the same paragraph, as well as a column called "right", constructed in the same manner for the text appearing to the right of the name.

To account for the fact that a name is often mentioned in identical or very similar left and or right contexts across multiple, not necessarily consecutive, revisions, our method groups "similar" left and right contexts. For each name and type of context, left and right, an undirected graph is created, in which each node represents a particular context in a particular revision. Two nodes are connected if the Levenshtein distance similarity ratio of the represented contexts is at least 75. The exact value of this ratio is used as edge weight.^^[[2]](#footnote-2)^^ In a final step, the nodes are clustered based on the Louvain method for community detection,^^[[3]](#footnote-3)^^ yielding left and right context IDs, respectively.

| 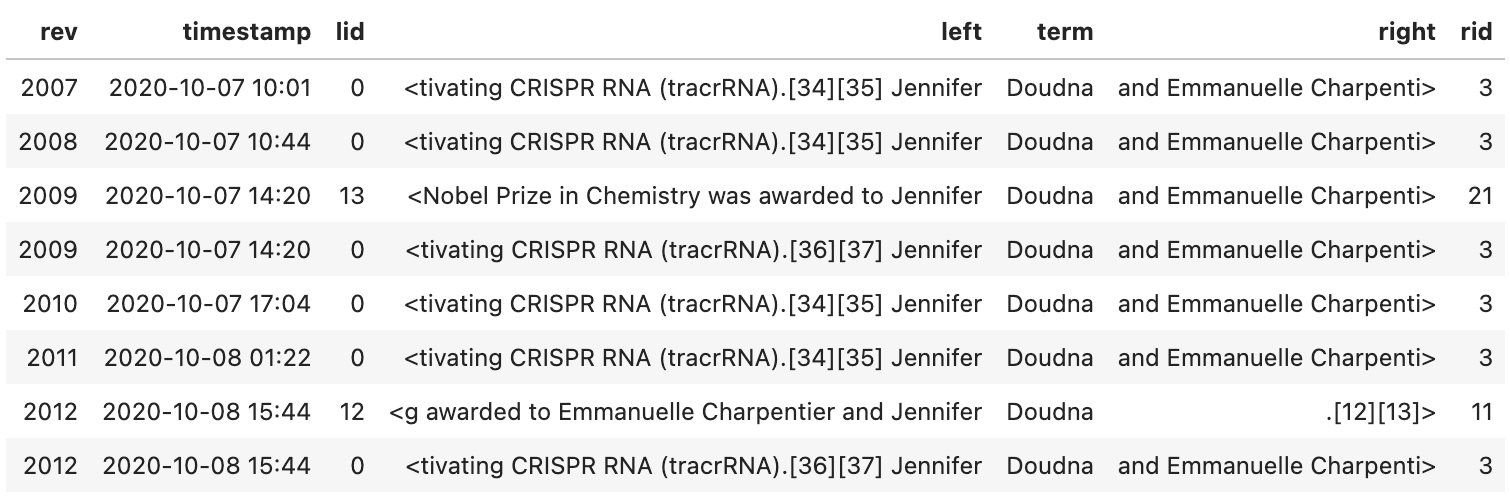 |
| --- |
| **Table S2**: Left and right contexts in which the name "Doudna" is mentioned during all six revisions of two successive days in October 2020: the day when the Nobel Prize for Chemistry was awarded to Jennifer Doudna and Emanuelle Charpentier, and the day after. |

The excerpt shown in Table S2 contains 8 rows, representing all individual mentions of Doudna over 6 consecutive revisions (ID 2007 through 2012) between October 8, 2020, the day when Charpentier and Doudna received the Nobel Prize, and the day after, October 9, 2020. We see that in each of the six revisions Doudna is mentioned in a reoccurring context in connection to "CRISPR RNA" to the left (lid: 0) and "Charpentier" to the right (lid: 3). In addition we see that Doudna is mentioned once next to "Nobel Prize […]" in revision ID 2009 (lid: 13), and again once next to "awarded to Emmanuelle Charpentier […]" in revision ID 2012 (lid: 12). This is because Doudna was linked to the Nobel Prize in revision ID 2009, unlinked again in revision ID 2010, and re-linked in revision ID 2012, albeit in a significantly dissimilar context. The "rid" in the third row is 21 instead of 3 as in the rows above and below, which seem to read exactly the same because we only see abbreviations of the 100 character contexts here. If expanded, the right context in the third row is actually very different from the ones in the rows above and below.

# Additional Information on Reported Controversies

The following information enriches the analysis in the main article's "Authorship and Recognition" section and the "Priority and Differentiation" section, respectively.

## Authorship and Recognition

Table S3 shows a series of (mainly) Type 2 name edits through which editors negotiated the micro-notability of co-authors of a paper providing experimental evidence that CRISPR works as an adaptive immune system

| Date | Edit | Type | Comment (Editor) |
| --- | --- | --- | --- |
| 2013-11-06 | **In 2007 Barrangou, Horvath and others showed that they could alter the resistance of Streptococcus thermophilus to phage attack with spacer DNA.[SOURCE]** | Type 1  insertion | lots of new stuff (Editor S1) |
| 2014-06-25 | In 2007 Barrangou, Horvath (food industry scientists at Danisco) and ~~others~~ **Moineau's** **group at Université Laval (Canada)** showed that they could alter the resistance of Streptococcus thermophilus to phage attack with spacer DNA.[SOURCE] | Type 2  insertion | →‎‎History (Editor 2) |
| 2016-06-22 | In 2007, Barrangou, Horvath**, and other** ~~(~~food industry scientists at Danisco~~), and Moineau's group at Université Laval (Canada) showed that they could use spacer DNA to alter the resistance of~~ **provided evidence that CRISPR-Cas immunity was adaptive. They found that a CRISPR region in** 'Streptococcus thermophilus' ~~to phage attack~~ **acquired spacers from DNA of infecting bacteriophage. Resistance to phage was manipulated by adding and deleting spacers whose sequence matched those found in the phages tested**. | Type 2 deletion,  Type 3 edit | →‎History: Expanded the single sentence paragraph describing the Danisco study (Editor 11) |
| 2016-10-29 | In 2007, Barrangou, Horvath, and ~~other food industry scientists~~ **colleagues** at Danisco **as well as Moineau's lab at the University Laval** provided the first experimental evidence that CRISPR was an adaptive immune system [...] | Type 2 insertion | Historical revisionism… (Editor 3) |
| 2016-10-29 | In 2007~~, Barrangou, Horvath, and colleagues at Danisco as well as Moineau's lab at the University Laval provided~~ the first experimental evidence that CRISPR was an adaptive immune system **was published** [...] | Type 2  deletion | →‎History: we don't care about this attribution (Editor 4) |
| 2016-10-29 | In 2007**, Barrangou, Horvath, and colleagues at Danisco as well as Moineau's lab at the University Laval** provided the first experimental evidence that CRISPR was an adaptive immune system ~~was published~~ [...] | Type 2  insertion | lol - "we" as you ! But that's OK (Editor 3) |
| 2016-10-29 | In 2007~~, Barrangou, Horvath, and colleagues at Danisco as well as Moineau's lab at the University Laval provided~~ the first experimental evidence that CRISPR was an adaptive immune system **was published** [...] | Type 2  deletion | Undid revision 746842119 by Editor 3 (talk) unsourced, see also WP:PROMO policy (Editor 4) |

**Table S3**: Name edits through which editors negotiated the micro-notability of co-authors of a paper providing experimental evidence that CRISPR works as an adaptive immune system.

## Priority Disputes

Table S4 shows a series of Type 1, 2, and 3 name edits in which editors negotiate micro-notability in relation to the question of who invented the genetic scissors.

| **Date** | **Edit** | **Type** | **Comment (Editor)** |
| --- | --- | --- | --- |
| 2013-11-12 | **CRISPR was first shown to work in human cells by George Church at Harvard University.[SOURCE]** | Type 1  insertion | →‎Discovery of CRISPR (Editor S2) |
| 2014-02-05 | CRISPR was first shown to work in human cells by **Feng Zhang of the Broad Institute[SOURCE] and** George M. Church**'s group** at Harvard University. | Type 2 insertion  Type 3 edit | →‎History (Editor S3) |
| 2014-03-14 | CRISPR was first shown to work as a genome engineering/ editing tool in human cell culture by **~~Feng Zhang's group at the Broad Institute[13]~~** **Jennifer Doudna in 2012 (SOURCE)** and George M. Church's group at Harvard University.[SOURCE] | Type 2 insertion  Type 2 deletion | [no comment provided] (Editor S4) |
| 2014-04-13 | CRISPR was first shown to work as a genome engineering/editing tool in human cell culture by ~~Jennifer Doudna in~~ 2012 ~~(SOURCE) and George M. Church's group at Harvard University~~.[SOURCES] | Type 2 deletion | consol refs (Editor S1) |
| 2016-04-26 | "​CRISPR was first shown to work as a genome engineering/editing tool in ~~human~~ **bacterial** cell culture in 2012 **by Jennifer Doudna and Emmanuelle Charpentier**.[SOURCES] | Type 2 insertion  Type 3 edit | →History: The cited article describes gene editing in bacterial cell culture system, not in human cell culture (Editor S5) |
| 2016-04-26 | [...Doudna/Charpentier as above…] **Feng Zhang and colleagues first described genome editing in human and mouse cell culture using CRISPR/Cas Systems.[SOURCES]** | Type 1 insertion | →‎History: Added Feng Zhang's work and citation (Editor S5) |
| 2016-05-01 | ~~CRISPR was first shown to work as a genome engineering/editing tool in bacterial cell culture in 2012 by Jennifer Doudna and Emmanuelle Charpentier.[SOURCES].~~ Feng Zhang and colleagues first described genome editing in human and mouse cell culture using CRISPR/Cas Systems.[SOURCES]  [...]  **In 2012, Jennifer Doudna and Emmanuelle Charpentier first showed CRISPR to work as a genome-engineering and -editing tool in bacterial cell cultures.[SOURCE]** | Type 1 deletion  Type 1 insertion | Editorial changes; e.g., no hyphen in unit modifier preceding a noun and whose first word is an adverb ending in -ly; better sources need in a couple of instances (explanatory note included). (Editor S6) |
| 2016-06-16 | [...Zhang as above…]  [...]  In 2012, Jennifer Doudna and Emmanuelle Charpentier ~~first showed CRISPR to work as a genome-engineering and -editing tool in bacterial cell cultures.[SOURCE]~~ **reengineered the natural four-component Cas9 endonuclease into a more manageable two-component system consisting of the Cas9 protein and a small RNA molecule. They also showed that the artificial Cas9 system could be programmed to target any sequence in DNA for cleavage.[SOURCE] This technological advance has fueled efforts to edit genomes with CRISPR-Cas9[SOURCE]** |  | ‎→Cas9: Added a review article that describes the importance of the paper by Doudna & Charpentier (Editor 11) |
| 2016-06-16 | ~~Feng Zhang and colleagues first described genome editing in human and mouse cell culture using CRISPR/Cas Systems.[SOURCES]~~  [...]  [...Doudna/Charpentier as above…]  [...]  **Feng Zhang and colleagues first described genome editing in human and mouse cell culture using CRISPR/Cas Systems.[SOURCES]** | Type 1  relocation | ‎→History: Moved paragraphs describing Cas9 applications into the "Cas9" subsection (Editor 11) |
| 2016-06-17 | [...Doudna/Charpentier as above…]  [...]  Feng Zhang**'s** and ~~colleagues first~~ **George Church's groups simultaneously** described genome editing in human ~~and mouse~~ cell culture**s** using CRISPR~~/~~**~~-~~**Cas Systems **for the first time**.[SOURCES] | Type 2 insertion  Type 3 edit | →‎Cas9: Church's group also demonstrated editing of human cell cultures. Zhang & Church papers were even published back-to-back in Science. Removed patent as source and added review (even Zhang writes that Church published their work simultaneously!) (Editor 11) |

**Table S4**: Name edits in which editors negotiate micro-notability in relation to the question of who invented the genetic scissors.

# Additional Analyses of Data

## Researcher Names in the Historical Accounts Corpus, Compared To Wikipedia

We systematically compared the occurrences and co-occurrences of names in our historical accounts corpus to the names found in the two Wikipedia articles, C1 and C2. Since there was no space to present this analysis in the main article, we present it here instead.

Table S5 lists the names of CRISPR researchers found in any of the 27 historical accounts, and whether or not the name is found in the Wikipedia articles "CRISPR" (C1) or "CRISPR gene editing" (C2).

| Name | C1/C2 | # Accounts | Accounts (detail) |
| --- | --- | --- | --- |
| Adli | not found | 1 | Adli 2018 |
| Anderson | not found | 1 | Riordan et al. 2015 |
| Barrangou | C1, C2 | 8 | Han and She 2017; Lander 2016; Lau and Davie 2017; Marraffini 2015; Mojica and Montoliu 2016; Mojica and Rodriguez-Valera 2016; Morange 2015a; Pennisi 2013 |
| Belmonte | not found | 1 | Liang et al. 2017 |
| Bolotin | C1 | 6 | Broad Institute 2015; Han and She 2017; Ishino et al. 2018; Lander 2016; Lau and Davie 2017; Mojica and Garret 2013 |
| Bondy-Demony | not found | 1 | Han and She 2017 |
| Brouns | C1 | 3 | Han and She 2017; Marraffini 2015; Mojica and Montoliu 2016 |
| Carte | not found | 1 | Han and She 2017 |
| Charpentier | C1, C2 | 13 | Adli 2018; Broad Institute 2015; Chen et al. 2020; Hsu et al. 2014; Javed et al. 2018; Lander 2016; Ledford 2016b; Ledford and Callaway 2020; Liang et al. 2017; Mojica and Montoliu 2016; Morange 2015b; Pennisi 2013; Sherkow 2015 |
| Chiarle | not found | 2 | Liang et al. 2017; Riordan et al. 2015 |
| Cho | not found | 1 | Lau and Davie 2017 |
| Church | C1, C2 | 8 | Broad Institute 2015; Chen et al. 2020; Lander 2016; Ledford 2016a; Ledford 2016b; Ledford and Callaway 2020; Liang et al. 2017; Pennisi 2013 |
| Cong | not found | 3 | Lau and Davie 2017; Ledford 2016a; Zhang 2019 |
| Del Bene | not found | 1 | Liang et al. 2017 |
| Deltcheva | not found | 1 | Han and She 2017 |
| Deng | not found | 1 | Han and She 2017 |
| Diez-Villasenor | not found | 1 | Mojica and Rodriguez-Valera 2016 |
| Doudna | C1, C2 | 11 | Chen et al. 2020; Hsu et al. 2014; Javed et al. 2018; Lander 2016; Ledford 2016a; Ledford 2016b; Ledford and Callaway 2020; Liang et al. 2017; Mojica and Montoliu 2016; Pennisi 2013; Sherkow 2015 |
| Firth | not found | 1 | Lau and Davie 2017 |
| Frew | not found | 1 | Riordan et al. 2015 |
| Fuji | not found | 1 | Riordan et al. 2015 |
| Gao | not found | 1 | Pennisi 2013 |
| Garneau | not found | 2 | Han and She 2017; Riordan et al. 2015 |
| Gasiunas | C1 | 1 | Ledford 2016a |
| Gersbach | C1 | 1 | Pennisi 2013 |
| Haft | C1 | 1 | Riordan et al. 2015 |
| Hale | not found | 1 | Han and She 2017 |
| Hayashi | not found | 1 | Lau and Davie 2017 |
| Horvath | C1, C2 | 7 | Adli 2018; Broad Institute 2015; Hsu et al. 2014; Lander 2016; Mojica and Montoliu 2016; Morange 2015a; Pennisi 2013 |
| Hotta | not found | 1 | Riordan et al. 2015 |
| Huang | not found | 2 | Liang et al. 2017; Riordan et al. 2015 |
| Ishino | C1 | 3 | Ishino et al. 2018; Lau and Davie 2017; Riordan et al. 2015 |
| Jacks | not found | 1 | Liang et al. 2017 |
| Jaenisch | not found | 3 | Mojica and Montoliu 2016; Morange 2015b; Pennisi 2013 |
| Jansen | C1 | 7 | Chen et al. 2020; Han and She 2017; Hsu et al. 2014; Lau and Davie 2017; Ledford and Callaway 2020; Mojica and Garret 2013; Riordan et al. 2015 |
| Jiankui | C1, C2 | 1 | Ledford and Callaway 2020 |
| Jinek | C1 | 6 | Adli 2018; Doudna and Charpentier 2014; Lau and Davie 2017; Ledford 2016a; Pennisi 2013; Riordan et al. 2015; |
| Joung | not found | 2 | Liang et al. 2017; Pennisi 2013 |
| Kim | not found | 1 | Liang et al. 2017 |
| Komor | not found | 1 | Adli 2018 |
| Koonin | C1 | 5 | Broad Institute 2015; Han and She 2017; Hsu et al. 2014; Ishino et al. 2018; Lander 2016; Lau and Davie 2017; Ledford and Callaway 2020; Mojica and Garret 2013; Mojica and Montoliu 2016; Mojica and Rodriguez-Valera 2016; Riordan et al. 2015 |
| Lai | not found | 1 | Liang et al. 2017 |
| Lander | not found | 1 | Liang et al. 2017 |
| Li | not found | 1 | Lau and Davie 2017 |
| Liu | not found | 2 | Chen et al. 2020; Liang et al. 2017 |
| Ma | not found | 1 | Riordan et al. 2015 |
| Makarova | not found | 4 | Han and She 2017; Ishino et al. 2018; Mojica and Garret 2013; Riordan et al. 2015 |
| Mali | not found | 1 | Ledford 2016a |
| Marraffini | C1 | 6 | Broad Institute 2015; Han and She 2017; Lander 2016; Ledford and Callaway 2020; Marraffini 2015; Mojica and Montoliu 2016; |
| Mashimo | not found | 1 | Liang et al. 2017 |
| Moineau | C1 | 4 | Broad Institute 2015; Hsu et al. 2014; Lander 2016; Mojica and Rodriguez-Valera 2016 |
| Mojica | C1 | 11 | Broad Institute 2015; Han and She 2017; Hsu et al. 2014; Ishino et al. 2018; Lander 2016; Lau and Davie 2017; Ledford and Callaway 2020; Mojica and Garret 2013; Mojica and Montoliu 2016; Mojica and Rodriguez-Valera 2016; Riordan et al. 2015; |
| Morgan | not found | 1 | Adli 2018 |
| Nakata | not found | 3 | Adli 2018; Hsu et al. 2014; Mojica and Montoliu 2016 |
| Nishihara | not found | 1 | Liang et al. 2017 |
| Ohtsuka | not found | 1 | Liang et al. 2017 |
| Peng | not found | 1 | Han and She 2017 |
| Porteus | not found | 1 | Liang et al. 2017 |
| Pourcel | not found | 2 | Han and She 2017; Ishino et al. 2018 |
| Qi | not found | 1 | Pennisi 2013 |
| Ran | not found | 2 | Ledford 2016a; Zhang 2019 |
| Rauch | not found | 1 | Han and She 2017 |
| Ren | not found | 1 | Riordan et al. 2015 |
| Rodriguez-Valera | not found | 1 | Mojica and Montoliu 2016 |
| Sapranauskas | not found | 1 | Han and She 2017 |
| Šikšnys | C1, C2 | 8 | Adli 2018; Broad Institute 2015; Hsu et al. 2014; Lander 2016; Ledford 2016a; Ledford and Callaway 2020; Liang et al. 2017; Mojica and Montoliu 2016 |
| Sontheimer | C1 | 5 | Broad Institute 2015; Han and She 2017; Lander 2016; Marraffini 2015; Mojica and Montoliu 2016 |
| Stern | not found | 1 | Mojica and Garret 2013 |
| Takada | not found | 1 | Riordan et al. 2015 |
| Tang | C1 | 1 | Han and She 2017 |
| Tessier-Lavigne | not found | 1 | Liang et al. 2017 |
| Thomson | not found | 1 | Liang et al. 2017 |
| Vakoc | not found | 1 | Liang et al. 2017 |
| Van der Oost | C1 | 4 | Broad Institute 2015; Hsu et al. 2014; Ishino et al. 2018; Lander 2016 |
| Ventura | not found | 1 | Liang et al. 2017 |
| Vergnaud | C1 | 1 | Lander 2016 |
| Vogel | not found | 1 | Lander 2016 |
| Wang | not found | 2 | Lau and Davie 2017; Riordan et al. 2015 |
| Wolfe | not found | 1 | Liang et al. 2017 |
| Wu | not found | 1 | Riordan et al. 2015 |
| Zhang | C1, C2 | 11 | Broad Institute 2015; Chen et al. 2020; Lander 2016; Ledford 2016a; Ledford 2016b; Ledford and Callaway 2020; Mojica and Montoliu 2016; Pennisi 2013; Riordan et al. 2015; Sherkow 2015; Zhang 2019 |
| Zhen | not found | 1 | Riordan et al. 2015 |

**Table S5**: Names found in our corpus of historical accounts of the CRISPR innovation. Column "C1/C2" shows whether the name is found in the Wikipedia articles "CRISPR" (C1) or "CRISPR gene editing" (C2). Columns "Accounts" and "Accounts (detail)" indicate how many historical accounts and which ones mention a name.

Figure S1 further shows that the overlap of the 36 names mentioned in C1 or C2 (Output 2) and the 82 names mentioned in our historical accounts corpus is 24. The accounts corpus contains 58 names that do not appear in C1 or C2, while 12 names in C1 or C2 are not mentioned in the accounts corpus.

| **Figure S1**: "Overlapping Names"— The diagram shows the overlap between unique names of CRISPR researchers found in Wikipedia's account of the CRISPR innovation history (C1 or C2) and/or in the historical account corpus. | 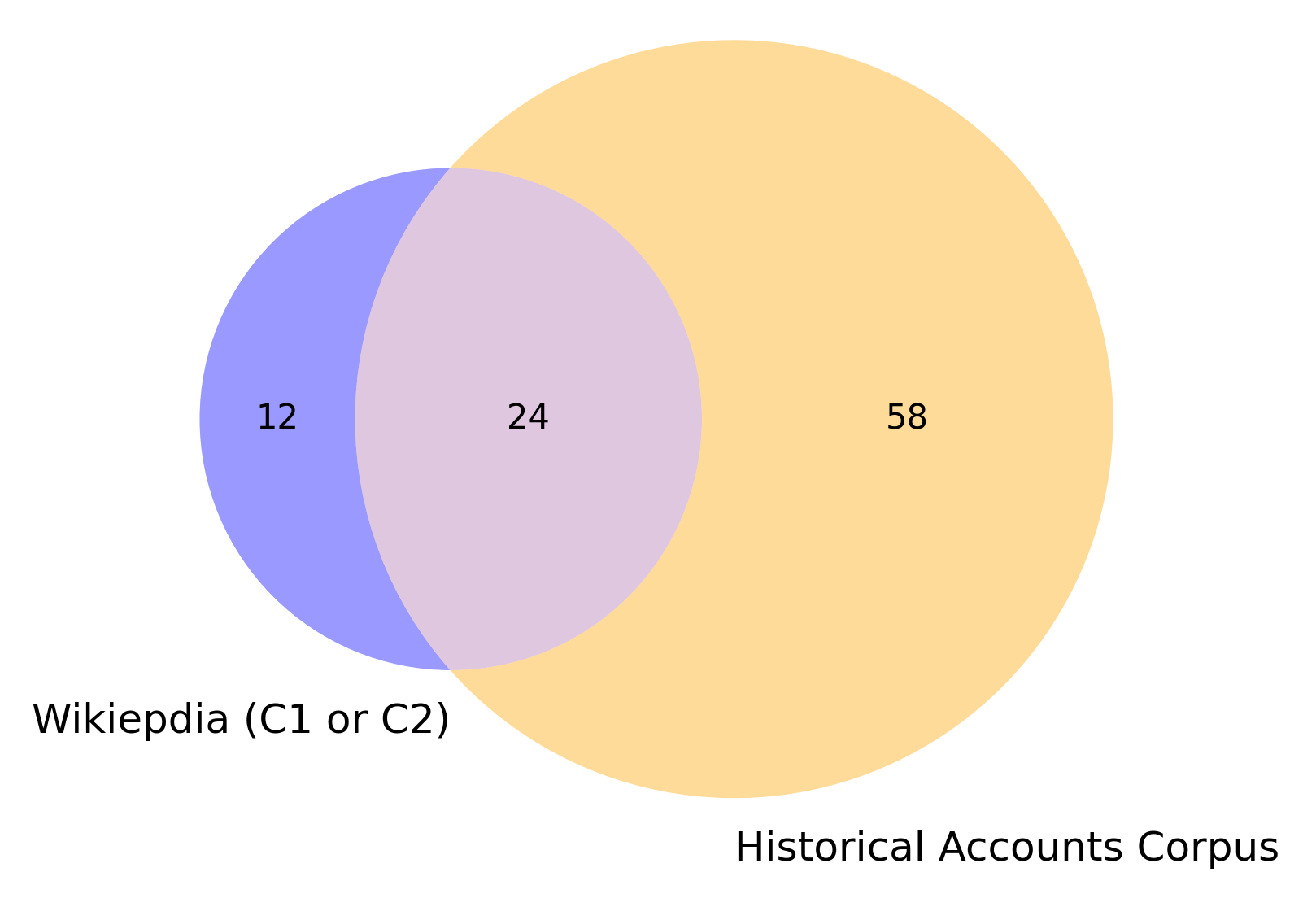 |
| --- | --- |

For each of the 82 names from the historical accounts corpus we also counted the number of individual accounts in which the name is mentioned (included in Table S5). To summarize these results, Table S3 lists three tiers of names distinguished and sorted by the number of times each name in the tier is mentioned in the historical accounts corpus. The table shows how many names in each of the three tiers are mentioned in C1 or C2 or in both. Tier 1 contains 17 names, each being mentioned in at least 4 historical accounts. 16 names in this tier are mentioned in C1, and seven are mentioned in C2. Tier 2 contains 13 names each of which is mentioned in two or three accounts. Only two of these names are found in C1, none is found in C2. Tier 3 contains 52 names each of which is only mentioned in one account. Six names are mentioned in C1, one is also mentioned in C2.

| Tier | Number of names tier | Mentioned in how many historical accounts | Mentioned in C1 | Mentioned in C2 |
| --- | --- | --- | --- | --- |
| 1 | 17 | 4 or more | 16 | 7 |
| 2 | 13 | 2 - 3 | 2 | 0 |
| 3 | 52 | 1 | 6 | 1 |

**Table S6**: Comparison of three tiers of names mentioned in the historical account corpus and in either C1 or C2 or in both.

While 24 of the 27 historical accounts mention names, even the most frequently mentioned name, Charpentier, only occurs in 13 accounts. Another peculiarity is that C1 mentions 6 names, all of which appear only in one historical account (Tier 3). Finally, we note that another 12 names mentioned by either C1 or C2 are not found in any of the historical accounts (Figure S1).

From this comparison we conclude that Wikipedia's account of notable CRISPR researchers in C1 and C2 is different from each historical account in our corpus, and it is also different from the union of all accounts in the corpus. We also conclude that the historical accounts converge only moderately in terms of the particular names they highlight.

This supports the argument made by others (Menking and Rosenberg, 2020; Moats, 2018; Wyatt et al., 2016) that Wikipedia's articles should not be mistaken as "the sum of all knowledge". Though Wikipedia's CRISPR articles are comprehensive and impressive, they remain a *distinct* account of which events and people should be considered relevant. Even if Wikipedia's collaborative and open approach to editing may counteract subjective bias and lead to more balanced articles, decisions still need to be made about what to include and what to leave out.

## Distribution of Editor Activity

Inspired by a comment of one of our reviewers, we created two extra plots, showing the sorted distribution of edits (Figure S2) and the normalized amount of edited text (Figure S3) for the top 150 editors. To calculate the normalized amount of edited text in Figure S3, we summed, for each editor, the relative number of bytes added or removed per revision, i.e the number of bytes weighted by the total number of bytes of the revisions.

The plot reveals that some of the editors who we observed to be quite dominant in fighting off contestants in controversies, such as Editors 5 and 13, also edited more actively than others. The relationship between edit activity and authoritative dominance should be studied further.


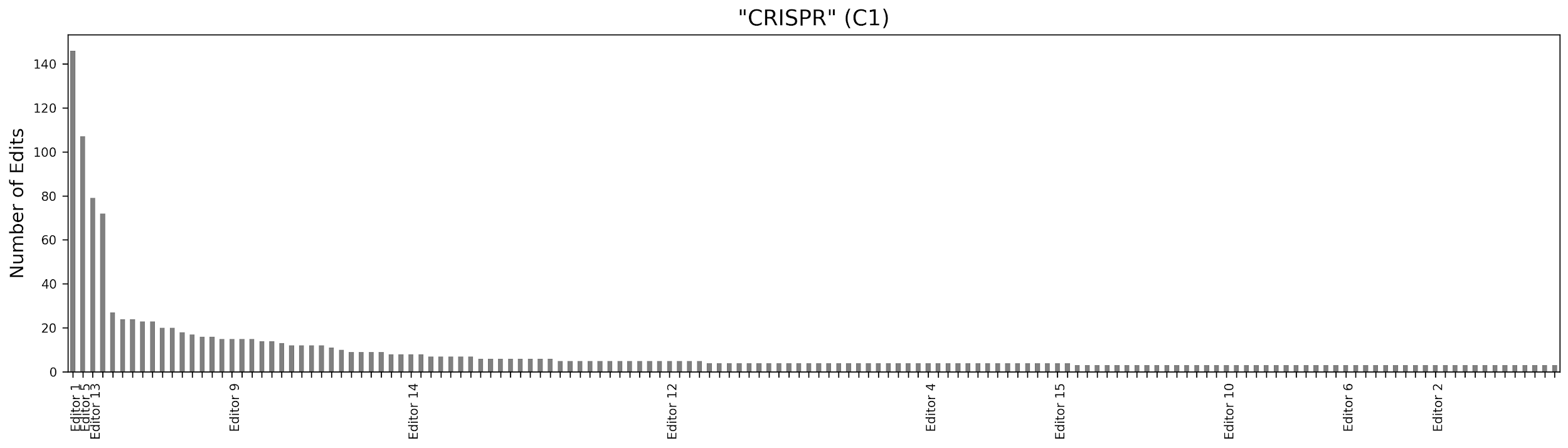


**Figure S2**: Distribution of the number of edits per editor in C1 for the top 150 editors.


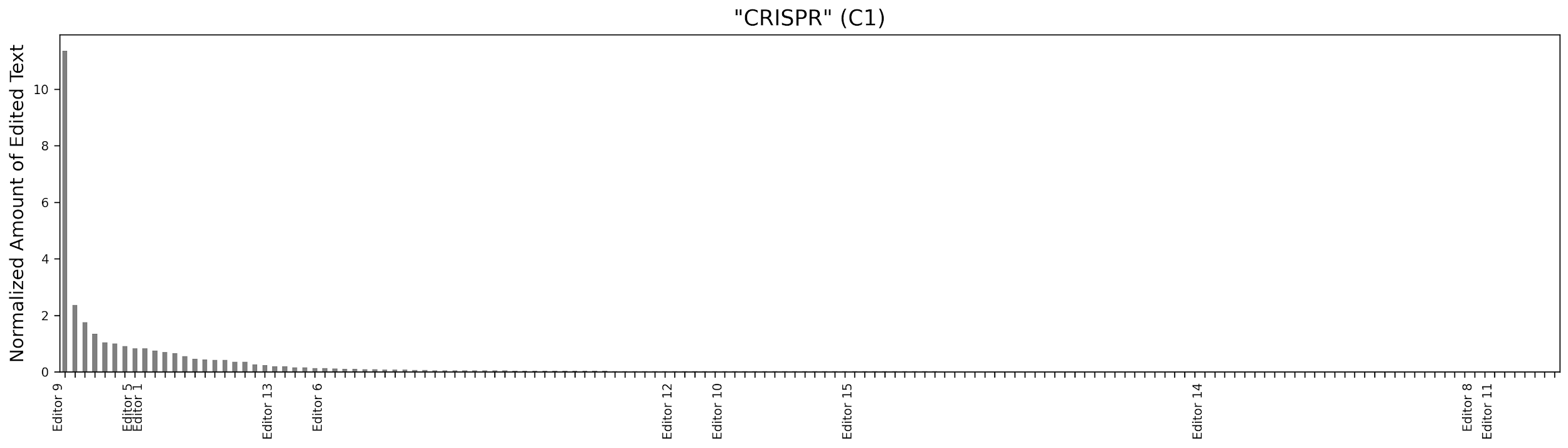


**Figure S3**: Distribution of the normalized amount of edited text per editor in C1 for the top 150 editors. The normalized amount of edited text was calculated by summing, for each editor, the relative number of bytes added or removed per revision, i.e the number of bytes weighted by the total number of bytes of the revisions.

1. Deletion log for C1: <https://en.wikipedia.org/w/index.php?title=Special:Log/delete&page=CRISPR>; deletion log for C2: <https://en.wikipedia.org/wiki/Special:Log?type=delete&user=&page=CRISPR+gene+editing> [↑](#footnote-ref-1)
2. We used the Levenshtein distance similarity ratio as implemented by the third-party Python library *fuzzywuzzy*. For any two strings, the ratio always ranges between 0 (totally unsimilar) and 100 (totally similar). [↑](#footnote-ref-2)
3. Blondel VD, Guillaume J-L, Lambiotte R, et al. (2008) Fast unfolding of communities in large networks. *Journal of Statistical Mechanics: Theory and Experiment* 2008(10): P10008. [↑](#footnote-ref-3)
